# A nationwide prospective cohort study on safety of the 17D-204 yellow fever vaccine during a vaccine shortage in Japan

**DOI:** 10.1093/jtm/taac070

**Published:** 2022-05-28

**Authors:** Yusuke Miyazato, Mari Terada, Mugen Ujiie, Sho Saito, Akinari Moriya, Masao Ando, Norio Ohmagari

**Affiliations:** Disease Control and Prevention Center, National Center for Global Health and Medicine, 1-21-1 Toyama, Shinjuku-ku, Tokyo 162-8655, Japan; Disease Control and Prevention Center, National Center for Global Health and Medicine, 1-21-1 Toyama, Shinjuku-ku, Tokyo 162-8655, Japan; Disease Control and Prevention Center, National Center for Global Health and Medicine, 1-21-1 Toyama, Shinjuku-ku, Tokyo 162-8655, Japan; Disease Control and Prevention Center, National Center for Global Health and Medicine, 1-21-1 Toyama, Shinjuku-ku, Tokyo 162-8655, Japan; Division of Quarantine and Sanitation, Chubu Airport Quarantine Office, Nagoya Quarantine Station, Nagoya 479-0881, Japan; Sendai Quarantine Station, Sendai 985-0011, Japan; Disease Control and Prevention Center, National Center for Global Health and Medicine, 1-21-1 Toyama, Shinjuku-ku, Tokyo 162-8655, Japan

## Abstract

In response to the vaccine shortage of yellow fever vaccine (YF-VAX) due to manufacturing delays, the unapproved 17D-204 YF-VAX was used as an investigator-initiated clinical trial in Japan. The vaccine was administered to 11 279 participants in 19 YF vaccination centres in Japan, and few serious adverse events were observed.

On 24 July 2017, Sanofi Pasteur, yellow fever vaccine (YF-VAX) manufacturer, announced a vaccine shortage caused by manufacturing delays.^1^ Consequently, some countries were forced to take compensatory measures. In the USA, expanded access was given to 17D-204 YF-VAX (Stamaril, Sanofi Pasteur SA, Lyon, France), under an investigational new drug protocol.^2^ Canada adopted fractional dosing of YF-VAX, administering 1/5th of the normal dose.^3^ In Japan, there is no regulatory system allowing emergency use of unlicensed medicines; 17D-204 YF-VAX was used as an alternative vaccine through an investigator-initiated clinical trial.^4^ As a clinical trial, the study objective was set upon trial initiation to collect safety information related to the 17D-204 YF-VAX injection.

We conducted a prospective, single-arm, multi-centre, interventional study to assess the safety of 17D-204 YF-VAX at 19 YF vaccination centres in Japan from October 2018 to August 2019. All eligible participants were injected with 17D-204 YF-VAX and observed for 30 min for immediate adverse events (AEs) and 30 days for non-immediate AEs. All participation was voluntary; however, during recruitment, no other YF-VAX product was available in Japan. Eligibility criteria^5^: participants at risk of YF, aged ≥9 months, were included in the study. The following participants were vaccinated after careful assessment by a physician: (i) pregnant and breastfeeding women, (ii) children aged 6–9 months, (iii) older persons aged ≥60 years, and asymptomatic human immunodeficiency virus (HIV)-infected patients with no evidence of impaired immune function. Exclusion criteria^5^: we excluded breastfeeding women who did not agree to discontinue breastfeeding for 14 days.

**Figure 1:**
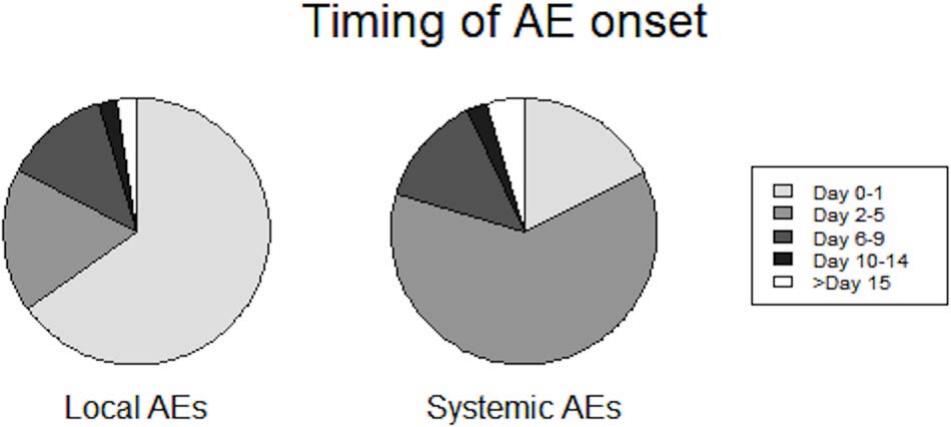
Timing of AE onset. For local AEs, 65.1% started on Days 0–1, while 61.8% of systemic AEs started on Days 2–5.

We collected information about immediate and non-immediate AEs from the study sites and participants by spontaneous patient-reported outcomes (PROs). Participants were asked to report any AE occurring within 30 days of vaccination via an electronic PRO system (ePRO) or paper PRO. PRO submission was not mandatory in the absence of any AEs. Determining true causality requires detailed information; thus, all AEs were included regardless of causality.

The study was approved by the Institutional Review Board of the National Center for Global Health and Medicine on 27 August 2018 (referral number: NCGM-C-003000-00). Written consent was obtained from participants upon study enrolment and vaccination.

**Table 1:** Summary of reportable and SAEs

Sex/age (y)	AE	Onset days after vaccination	Serious/reportable	Causality	Comment
M/63	Intestinal obstruction	36	Serious	Not related	On Day 36 after vaccination, the subject felt nauseous and visited a nearby clinic on Day 37. He complained of loss of appetite, burning in the chest and stomachache. On Day 38, he was admitted to a hospital. The subject had a history of abdominal surgery, and computed tomography showed bowel obstruction. He was diagnosed with adhesive bowel obstruction, which was treated conservatively and the patient recovered
M/22	Tonsillitis	29	Serious	Related/cannot be ruled out	The subject with no previous medical history presented with mild sore throat and fever appearing on Day 6 post-vaccination, which was judged to have been caused by the vaccination by the site physician. His symptoms resolved, but he then again experienced sore throat during travelling, from Day 29. After he had returned to Japan, he visited a clinic and was admitted for tonsillitis on Day 33. He was treated with antimicrobials. Despite this final diagnosis, the site physician maintained the causality assignment
M/49	Fever, myalgia, headache	6	Serious	Related/cannot be ruled out	The subject presented with fever, myalgia and headache on Day 6 after vaccination. Despite the use of antipyretics, he required hospitalization from Day 7 to 10 due to prolonged fever and malaise
F/60	Reactive polyarthritis	5	Serious	Related/cannot be ruled out	The subject with a history of cholecystectomy due to cholelithiasis but no other comorbidities presented with fever and headache on Day 1 post-vaccination. Pain and swelling developed in her knees on Day 5. Joint pain developed in her upper limb on Day 12 and her temperature was highest, at 39°C, on Day 24. On Day 27, her fever resolved but her joint pain did not resolve by Day 35; she also had difficulty in walking and was referred to a nearby hospital. After collagen disease including rheumatoid arthritis was ruled out at the orthopaedic department, she was diagnosed with reactive polyarthritis and treated with non-steroidal anti-inflammatory drugs and prednisolone. The dose of prednisolone was gradually tapered and finally all oral medications were discontinued on Day 210. Her walking ability was roughly restored and her hospital follow-up ended on Day 225
F/29	Pregnancy	−28	Reportable	Not related	The subject used an over-the-counter pregnancy kit and tested positive on Day 10 post-vaccination. Her pregnancy was confirmed on Day 13. According to her menstrual date, the start of pregnancy was calculated as Day −28 relative to vaccination. She did not have strong concern about the impact of 17D-204 YF-VAX on her pregnancy. No notable issues were detected during the pregnancy. She delivered a 2600 g baby by planned caesarean section at full term (37 weeks and 3 days). The baby was placed in an incubator for 2 days because of arrhythmia but was discharged as planned. The child is feeding well on mother’s milk and formula
F/25	Pregnancy	−32	Reportable	Not related	The subject used an over-the-counter pregnancy kit and tested positive on Day 3 post-vaccination. Her pregnancy was confirmed on Day 6. According to menstrual date, the start of the pregnancy was calculated as Day −32 relative to vaccination. Due to financial reasons, the subject and the father decided to end the pregnancy. They explained that injection of 17D-204 YF-VAX was not the reason for the abortion. Abortion surgery was performed on Day 9. Her post-operative condition was good, and the event was considered resolved
F/33	Breastfeeding	2	Reportable	Not related	The subject breastfed her child once because the baby did not eat baby food. No adverse event occurred to the baby

Onset time is counted from vaccination day (vaccination = Day 0). F, female; M, male.

Continuous variables were compared between groups using *t*-tests, and binary outcomes were compared using the chi-square test. Multivariate logistic regression analyses were performed to calculate the adjusted odds ratios (aORs) with 95% confidence intervals (CIs) for AE occurrence. Statistical significance was set at *P* < 0.05. Stata 17.0 (StataCorp, College Station, TX, USA) was used to conduct all statistical analyses.

Of the 11 284 candidates, 11 279 were vaccinated and were included in the analysis (Supplementary Figure 1). Vaccinees were predominantly Japanese (10 748 [95.3%] vs 531 [4.71%]), and nearly two-thirds of them were male (7147 [63.4%] vs 4132 [36.6%]) (Supplementary Table 1). There were 922 vaccinees requiring special consideration for vaccination, of which 922 were ≥60 years, and one also had asymptomatic HIV infection without evidence of impaired immune function. There were no infants <9 months or pregnant women in the study cohort. There were 22 infants aged ≥9 and <12 months.

AEs were observed in 696 participants (6.17%), of which 86 and 656 developed local and systemic AEs, respectively. Immediate AEs occurred in 32 participants. Spontaneous PRO was collected through ePRO from 543 (4.81%) participants. Among these AEs, fever was most frequently observed (424, 3.76%), followed by fatigue (367, 3.25%) and headache (254, 2.25%) (Supplementary Table 1-3). The results of the multivariate analyses showed that older age (>60 years) did not influence the odds of local AE development but was associated with higher odds of developing systemic AEs (aOR 1.34 [95% CI 1.03–1.73], *P =* 0.026) (Supplementary Table 4). The timing of onset of local and systemic AEs is shown in Figure 1: systemic AEs were observed later than local AEs. Most AEs were observed by Day 9: 95.3% for local and 92.9% for systemic AEs.

Reportable and serious AEs (SAEs), including pregnancy and breastfeeding, are shown in Table 1. Two pregnancies were confirmed within 30 days of vaccination; one participant delivered a baby by planned caesarean section, and the other terminated her pregnancy for financial reasons. One case of breastfeeding occurred within 14 days of vaccination. Overall, there were no apparent AEs due to vaccination during pregnancy or lactation. Other reportable events, including YEL-AND and YEL-AVD, did not occur. No anaphylactic reactions requiring hospitalization were observed, but three participants developed SAEs that may have been related to the vaccination (Table 1). Two of these SAEs (tonsillitis and reactive polyarthritis) were unexpected from the summaries of product characteristics.

In the USA, the Food and Drug Administration accepted the use of 17D-204 YF-VAX under an investigational new drug protocol in an Expanded Access Program.^2^ Expanded access involves using an investigational new drug outside of a clinical trial in patients with serious disease or conditions.^6^ This system is more practical than clinical trials and can easily be adapted to emergency situations, but there is no such system in Japan. Our experience in a study that required great time and effort would support a policy change that would allow for more flexible practice.

This study had several limitations. First, the frequency of AEs was markedly lower than that in previous studies.^7–10^ We obtained response data only from 543 (4.81%) vaccinees. This low reporting rate is probably because priority was placed on the feasibility of vaccinating all travellers who required vaccination within a short time, and some travellers may have had difficulty accessing the electronic PRO system. Our results may have been affected by the non-response bias, which may have contributed to the lower incidence of AEs compared to that reported in the literature. Second, we could not collect data on the simultaneous administration of other vaccines and symptoms caused by vaccination other than for YF may have been included as AEs.

In conclusion, we completed a large-scale YF vaccination program in 10 months, using a vaccine not approved in Japan as a clinical study in response to the suspension of distribution of the approved YF-VAX. There were few reports of SAEs related to this vaccine, and it has been shown to be generally safe for Japanese individuals. This study was conducted because Japan does not have a system similar to the Expanded Access Program in the USA; however, it is considered a significant example of how to respond to emergencies, such as a delay in the supply of essential medicines.

## Supplementary Material

Supplementary_Figure_1_taac070

Supplementary_material_cleaned_taac070
