# Supplementary figures and images for: A nationwide prospective cohort study on safety of the 17D-204 yellow fever vaccine during a vaccine shortage in Japan
Source: J Travel Med. 2022 May 28;30(2):taac070. doi: 10.1093/jtm/taac070 (PMC10075058; doi:10.1093/jtm/taac070)

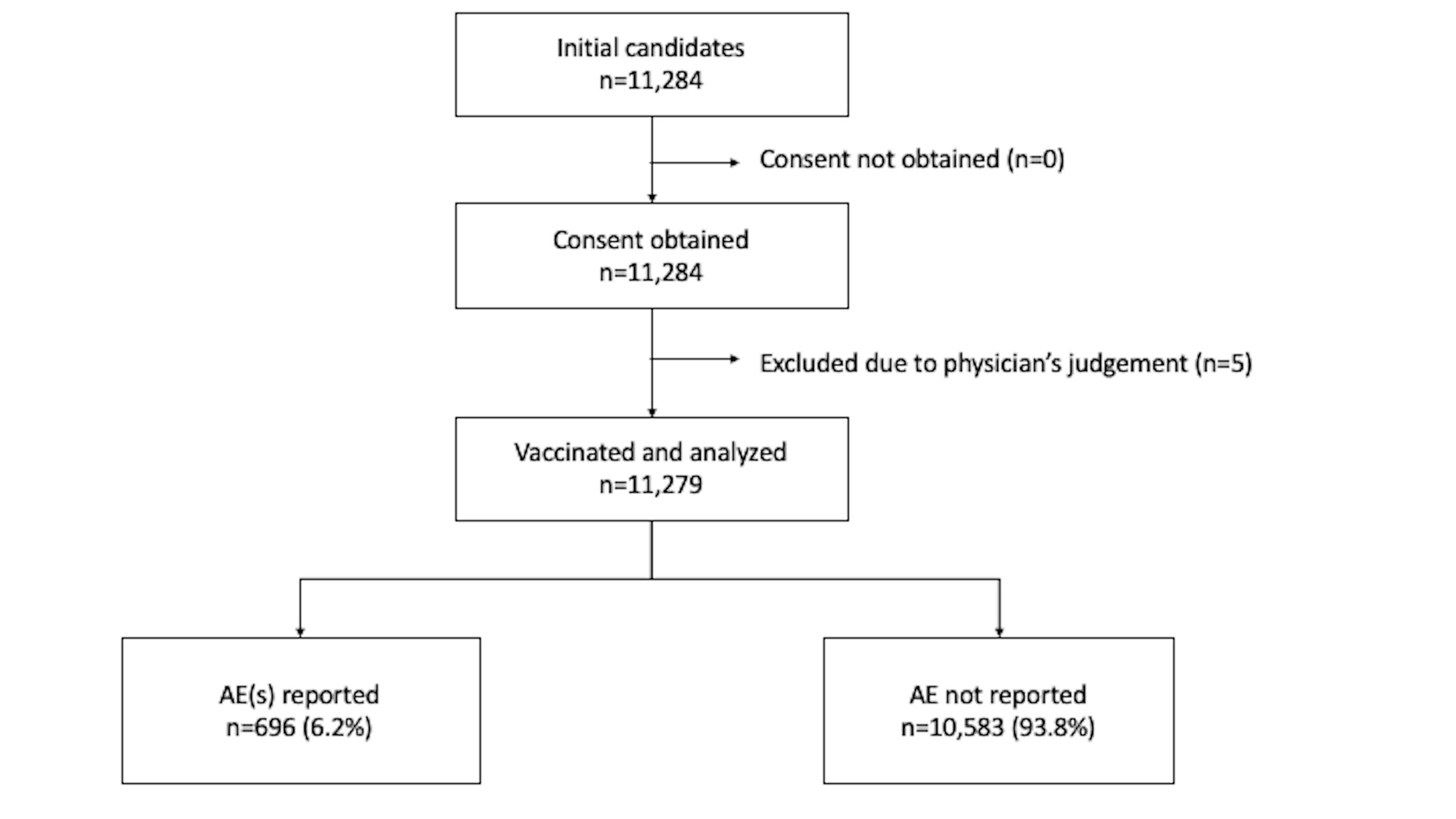

Supplement: Supplementary_Figure_1_taac070 [file supplementary_figure_1_taac070.zip › Supplementary_Figure_1_taac070.tiff]
