# Supplementary material for: A nationwide prospective cohort study on safety of the 17D-204 yellow fever vaccine during a vaccine shortage in Japan
Source: J Travel Med. 2022 May 28;30(2):taac070. doi: 10.1093/jtm/taac070 (PMC10075058; doi:10.1093/jtm/taac070)
Supplement: Supplementary_material_cleaned_taac070 [file supplementary_material_cleaned_taac070.docx]

**Supplementary material**

**Supplementary Table 1.** Characteristics of the vaccinees (n=11,279)

|  |  | Total | Male | Female |
| --- | --- | --- | --- | --- |
| **Age (years)** | | | | |
|  | Mean ± SD | 36.5 ± 15.1 | 37.5 ± 14.9 | 34.6 ± 15.4 |
|  | Median [IQR] | 34 [25, 47] | 36 [26, 48] | 31 [24, 45] |
| **Sex (n, %)** | | − | 7147 (63.4%) | 4132 (36.6%) |
| **Nationality (n, %)** | | | | |
|  | Japanese | 10748 (95.3%) | 6866 (96.1%) | 3882 (93.4%) |
|  | Other | 531 (4.71%) | 281 (3.93%) | 250 (6.05%) |
| **AE (n, %)** | | | | |
|  | Within 30 min | 32 (0.28%) | 18 (0.25%) | 14 (0.34%) |
|  | Any AE | 696 (6.17%) | 504 (7.05%) | 192 (4.65%) |
|  | Local AE | 86 (0.76%) | 29 (0.41%) | 57 (1.40%) |
|  | Systemic AE | 667 (5.91%) | 487 (7.00%) | 169 (4.16%) |
| **Age category (n, %)** | | | | |
|  | ≥ 60 | 922 (8.17%) | 573 (8.02%) | 350 (8.47%) |
|  | < 60 | 10357 (91.8 %) | 6573 (92.0%) | 3782 (91.5%) |
| **Spontaneous report through ePRO (n, %)** | | | | |
|  | Submitted | 543 (4.81%) | 388 (5.43%) | 155 (3.75%) |
|  | Not submitted | 10736 (95.2%) | 6759 (94.6%) | 3977 (96.2%) |

Abbreviations: AE, adverse events; IQR, interquartile range; SD, standard deviation; ePRO, electronic patient-reported outcomes system.

**Supplementary Table 2.** Study population and adverse event occurrence by age group

|  | Total population | | Submitted PROs | | Local AEs | | Systemic AEs | |
| --- | --- | --- | --- | --- | --- | --- | --- | --- |
| Age (years) | N | % | N | %* | N | %* | N | %* |
| 0‒9 | 369 | 3.27 | 17 | 4.61 | 4 | 1.08 | 15 | 4.07 |
| 10‒19 | 597 | 5.29 | 15 | 2.51 | 6 | 1.01 | 19 | 3.18 |
| 20‒29 | 3197 | 28.3 | 74 | 2.31 | 15 | 0.50 | 104 | 3.25 |
| 30‒39 | 2706 | 24.0 | 136 | 5.03 | 23 | 0.85 | 176 | 6.50 |
| 40‒49 | 2043 | 18.1 | 138 | 6.75 | 19 | 0.93 | 163 | 7.98 |
| 50‒59 | 1445 | 12.8 | 103 | 7.13 | 11 | 0.76 | 119 | 8.24 |
| 60‒69 | 720 | 6.38 | 54 | 7.50 | 6 | 0.83 | 64 | 8.89 |
| > 70 | 202 | 1.79 | 6 | 2.97 | 2 | 0.99 | 7 | 3.47 |
| Total | 11279 | 100 | 543 | 4.81 | 86 | 0.76 | 667 | 5.91 |

*Percentage of subjects who submitted PROs and reported local AEs or systemic AEs indicate the rate within each age group.

Abbreviations: AEs, adverse events; PROs, patient-reported outcomes; N, number of participants.

**Supplementary Table 3.** Number and percentage of solicited and unsolicited AEs.

| **Type of AE** | **Frequency of AEs** | |
| --- | --- | --- |
|  | **N** | **%** |
| **Local AEs** | **86** | **0.76** |
| **General symptoms** | **588** | **5.21** |
| Fever | 424 | 3.76 |
| Fatigue | 367 | 3.25 |
| Lymphadenopathy | 22 | 0.20 |
| Other | 32 | 0.28 |
| **Musculoskeletal symptoms** | **315** | **2.79** |
| Myalgia | 220 | 1.95 |
| Arthralgia | 217 | 1.92 |
| Other | 12 | 0.11 |
| **Skin symptoms** | **69** | **0.61** |
| Pruritus | 46 | 0.41 |
| Rash | 34 | 0.30 |
| Hot Flashes | 2 | 0.02 |
| **Neurologic Symptoms** | **361** | **3.20** |
| Headache | 254 | 2.25 |
| Drowsiness | 71 | 0.63 |
| Paresthesia | 52 | 0.46 |
| Dizziness | 46 | 0.41 |
| Vagovagal reflex | 10 | 0.09 |
| Other | 14 | 0.12 |
| **Gastrointestinal Disorders** | **178** | **1.58** |
| Diarrhea | 98 | 0.87 |
| Appetite Loss | 62 | 0.55 |
| Abdominal Pain | 40 | 0.35 |
| Nausea | 39 | 0.35 |
| Vomiting | 19 | 0.17 |
| Other | 3 | 0.03 |
| **Ear, Nose, Throat symptoms** | **51** | **0.45** |
| Rhinitis | 31 | 0.27 |
| Sore throat | 16 | 0.14 |
| Other | 13 | 0.12 |
| **Others** | **24** | **0.21** |
| **Reportable Events** | **3** | **0.03** |

Names in boldface indicate system organ class (SOC), and those in plain letters indicate AE names.

Within each SOC, AEs with fewer than 10 reports were added together in “other,” excluding hot flashes. The number of AEs does not add up to the number of AEs in SOC, as some participants experienced multiple symptoms within the SOC.

Abbreviations: AEs, adverse events; N, number of participants; SOC, system organ class.

**Supplementary Table 4.** Results of multivariate logistic regression analysis for all, local, and systemic adverse events.

| ALL AEs | AOR | 95%CI | p-value |
| --- | --- | --- | --- |
| Age ≥ 60 years | 1.34 | 1.04‒1.72 | 0.023 |
| Female | 0.64 | 0.54‒0.77 | <0.001 |
| Local AEs |  |  |  |
| Age ≥ 60 years | 1.16 | 0.56‒2.42 | 0.691 |
| Female | 4.26 | 2.61‒6.94 | <0.001 |
| Systemic AEs |  |  |  |
| Age ≥ 60 years | 1.34 | 1.03‒1.73 | 0.026 |
| Female | 0.57 | 0.47‒0.68 | <0.001 |

Abbreviations: AOR, adjusted odds ratio; AEs, adverse events.

Individuals aged ≥ 18 years were included in the analyses.

**Supplementary Figure legends**

**Supplementary Figure 1.** Process of participant enrollment in the study.

Five were excluded, after obtaining consent, due to the physician’s judgment. Of those, two had no plans to travel to an endemic area, one did not attend the hospital on the vaccination day, one had intractable psoriasis vulgaris, and one had serologically proven egg allergy. We issued waiver forms for patients with psoriasis and egg allergy.
